# Effects of GH/IGF on the Aging Mitochondria

**DOI:** 10.3390/cells9061384

**Published:** 2020-06-02

**Authors:** Sher Bahadur Poudel, Manisha Dixit, Maria Neginskaya, Karthik Nagaraj, Evgeny Pavlov, Haim Werner, Shoshana Yakar

**Affiliations:** 1 David B. Kriser Dental Center, Department of Molecular Pathobiology, New York University College of Dentistry New York, NY 10010–4086, USA; sbp4@nyu.edu (S.B.P.); dixitm01@nyu.edu (M.D.); mn2452@nyu.edu (M.N.); ep37@nyu.edu (E.P.); 2 Department of Human Molecular Genetics and Biochemistry, Sackler School of Medicine, Tel Aviv University, Tel Aviv 69978, Israel; mailkartz@gmail.com (K.N.); hwerner@tauex.tau.ac.il (H.W.)

**Keywords:** mitochondria, growth hormone, insulin-like growth factor-1, aging, oxidative stress, senescence

## Abstract

The mitochondria are key organelles regulating vital processes in the eukaryote cell. A decline in mitochondrial function is one of the hallmarks of aging. Growth hormone (GH) and the insulin-like growth factor-1 (IGF-1) are somatotropic hormones that regulate cellular homeostasis and play significant roles in cell differentiation, function, and survival. In mammals, these hormones peak during puberty and decline gradually during adulthood and aging. Here, we review the evidence that GH and IGF-1 regulate mitochondrial mass and function and contribute to specific processes of cellular aging. Specifically, we discuss the contribution of GH and IGF-1 to mitochondrial biogenesis, respiration and ATP production, oxidative stress, senescence, and apoptosis. Particular emphasis was placed on how these pathways intersect during aging.

## 1. Overview of the growth hormone (GH)/insulin-like growth factor (IGF ) Axis

Growth hormone (GH) and the insulin-like growth factor-1 (IGF-1) are part of the somatotropic hypothalamic-pituitary axis that regulates somatic growth and aging. As such, GH and IGF-1 peak during puberty to support lean and fat mass gain as well as to enhance skeletal acquisition and linear growth. During aging, GH and IGF-1 levels are significantly reduced, a state termed somatopause. Somatopause associates with many pathologies, such as osteopenia [1], sarcopenia [2], cardiovascular disorders [3,4], and more. For the sake of this review, it is important to distinguish between congenital somatopause, which results from germline mutations in components of the GH/IGF-1 axis (resulting in life-long epigenetic changes), and age-induced somatopause, which refers to the natural decline in GH and IGF-1 levels during aging.

**Figure 1:**
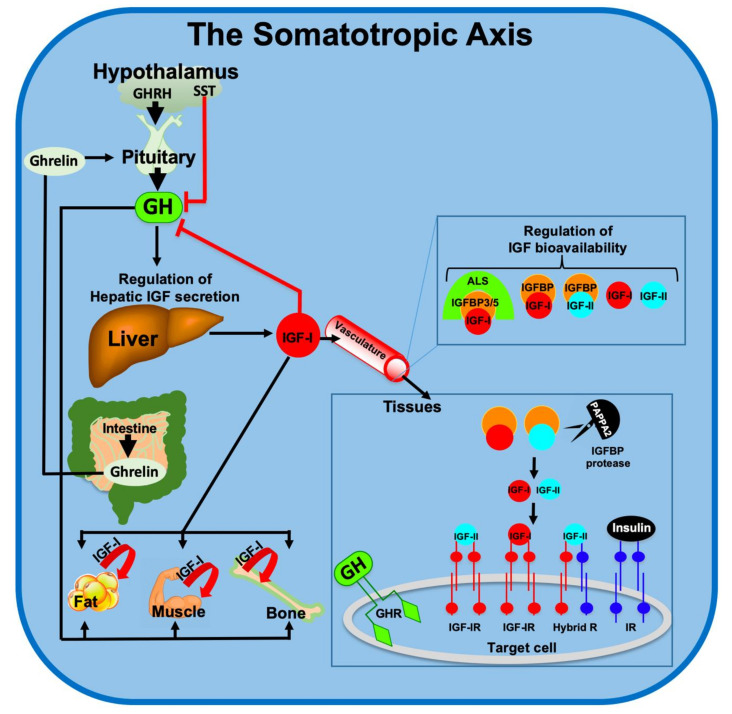
Schematic summary of the major molecules composing the somatotropic axis. GHRH—Growth hormone-releasing hormone, SST—Somatostatin, GH—Growth hormone, GHR—Growth hormone receptor, IGF—Insulin-like growth factor, IGFBP—Insulin-like growth factor binding protein, IGF-IR—insulin like growth factor receptor-1, IR—Insulin receptor, ALS—Acid labile subunit, PAPPA2—Pregnancy-associated plasma protein-A2.

The GH/IGF-1 axis includes the hypothalamic GH-releasing hormone (GHRH) and its receptor (GHRHR), expressed mainly in the pituitary (Figure 1) [5]. Upon activation, the GHRHR stimulates pituitary secretion of GH, which is released to the circulation and binds to its specific membrane receptor (GHR) on cells of different tissues. Among the many actions of GH in liver, its major role is stimulating the transcription of the IGF-1 gene, the IGF-binding proteins (IGFBPs) [6], and the acid labile subunit (ALS) that carry IGF-1 in circulation [7]. Other members of this axis are the proteases that release the IGF-1 from its complex with IGFBPs, pregnancy-associated plasma protein A (PAPP-A), and the PAPP-A2 [8]. The somatotropic axis is tightly regulated under normal physiological conditions. IGF-1 in serum provides a negative feedback to the pituitary to specifically inhibit GH secretion. Somatostatin (SST), secreted by the hypothalamus, inhibits several pituitary hormones including GH. In addition, SST binds to several SST-receptors (SSTR) on peripheral tissues to inhibit production of pancreatic (glucagon and insulin) and gastric (secretin and gastrin) hormones. Secretion of GH is also regulated by the gastrointenstinal hormone ghrelin. Ghrelin is one of the hormones that regulate food intake [9]. During hunger, ghrelin levels in circulation are increased and activate hypothalamic ghrelin receptor to initiate appetite. In addition, ghrelin acts as a GH secretagogue and can induce pituitary secretion of GH [10].

Receptors to GH and IGF-1 are present in virtually all cells. The IGF-1 receptor (IGF-1R) is a tyrosine kinase receptor that activates multiple pathways including (but not limited to) the Phosphoinositide-3-kinase (PI3K)/Protein kinase B (PKB or AKT), Ras/Raf/Mitogen-activated protein kinase (MAPK), and Shc [11]. These pathways are implicated in cellular growth, proliferation, differentiation, survival, metabolism, gene transcription, and protein translation. Apparently, the PI3K/AKT pathway is central to regulation of cell metabolism and cell fate (apoptosis), both of which involve mitochondrial function and integrity. The GHR is a cytokine-like receptor that, upon ligand binding, activates the Janus kinase 2 (JAK2)/Signal transducer and activator of transcription 5B (STAT5b) pathway to induce transcriptional activation of genes involved in cellular growth (among them is *Igf1*) and metabolic homeostasis [11].

Among other aspects of cellular homeostasis, GH and IGF-1 have important roles in mitochondrial function. Mitochondria participate in a wide range of processes with vital roles in cellular function. A key function of mitochondria is generation of adenosine triphosphate (ATP) from metabolic processing of carbohydrates, fats, and amino acids. In addition, mitochondria regulate other functions such as calcium homeostasis, cell death programming (apoptosis), inflammation, heat production, and other tissue-specific functions. Given their critical roles in the cell, it is not surprising that mitochondria are considered central regulators of aging. This review summarizes a few aspects of mitochondrial regulation by the GH/IGF-1 axis. We could not possibly include all of the studies that reported how GH/IGF-1 control mitochondrial function, including the effects of this axis on mitochondria in cancer cells (which can be found in [12,13,14]). Instead, our review focuses mostly on different aspects of aging and how GH/IGF-1 affects mitochondria in that context.

## 2. GH/IGF-1 Effects on Mitochondrial Biogenesis

Mitochondria are dynamic organelles. The number and size of mitochondria change according to the cellular metabolic and physiologic conditions. In addition, mitochondria abundance involves fusion (merging of two originally distinct mitochondria into one), fission (division of a single mitochondrion into two distinct mitochondria), and biogenesis. Mitochondrial biogenesis requires coupling of mitochondrial genome replication and fission.

**Figure 2:**
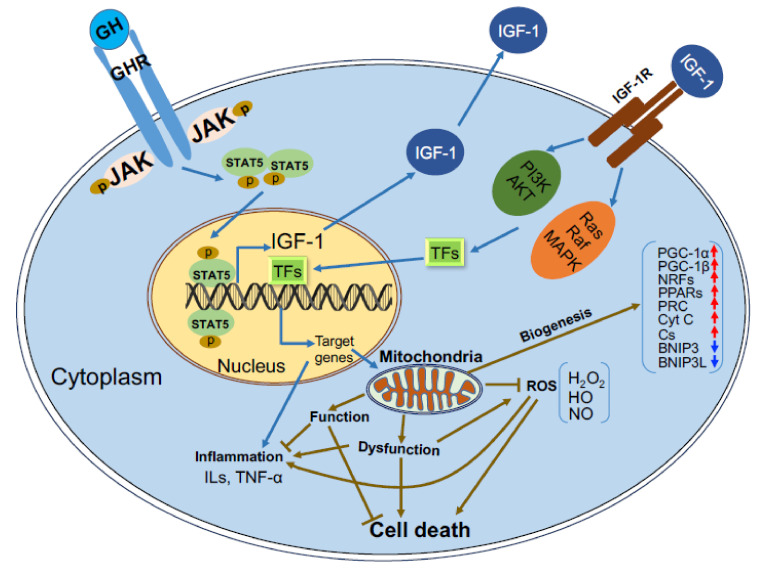
Schematic summary of the major effects of GH/IGF-1 on mitochondrial gene expression: Upon binding of GH to the GHR, the Janus kinase (JAK)-Signal transducer and activator of transcription 5 (STAT5) signaling pathway is activated, leading mostly to increases in IGF-1 transcription. Binding of IGF-1 to the tyrosine kinase IGF-1R stimulates several signaling pathways including the Phosphoinositide-3-kinase (PI3K)/Protein kinase B (PKB or AKT) and Ras/Raf/Mitogen-activated protein kinase (MAPK), involving phosphorylation and dephosphorylation of candidate proteins. This cascade leads to transcriptional activity of genes involved in mitochondrial biogenesis, control of Reactive oxygen species (ROS), cell survival (antiapoptotic), and genes involved in metabolism.

The mitochondrial genome (mtDNA) encodes 13 proteins, 22 transfer-RNAs, and two ribosomal-RNAs [15]. The 13 proteins encoded by mitochondria take part in oxidative phosphorylation (OXPHOS) including cytochrome b of complex III; ATP synthase subunits 6 and 8 of the F_0_ ATP-synthase complex; cytochrome c (Cyt c) oxidase subunits 1, 2, and 3; and six subunits of the nicotinamide adenine dinucleotide (NADH) dehydrogenase of complex I (ND1 through ND6). However, there are approximately 1500 proteins encoded by the nucleus which are necessary for mitochondrial biogenesis and function [16,17]. As such, replication of the mitochondrial genome is a complex mechanism that requires activation and translation of a series of nuclear encoded proteins. These mitochondrially targeted proteins are transported to the mitochondria via channels composed of transporters located at the outer mitochondrial membrane (TOMs) [18]. One of the proteins that plays key roles in mitochondrial biogenesis is peroxisome proliferator-activated receptor gamma coactivator 1 α (PGC1α). Activation of PGC1α leads to subsequent activations of the transcriptional regulators, nuclear respiratory factors (NRF1 and 2), and peroxisome proliferator-activated receptors (PPARs), which initiate transcription of nuclear genes involved in mitochondrial biogenesis and function (Figure 2).

The possible roles of the GH/IGF-1 axis in mitochondrial biogenesis were initially recognized in the early 1970s. Following injection of radiolabelled bovine GH (^125^I-bGH) to Sprague Dawley rats, radioactive signals were detected in the mitochondria, nuclei, and other cytoplasmic compartments of liver and kidney cells [19]. However, a later study found that, in fact, a very negligible amount of radiolabelled ^125^I-bGH gets transported to the mitochondria compared to other subcellular compartments, including microsomes, endosomes, lysosomes, and Golgi bodies [20], suggesting that GH does not act directly on mitochondria. Instead, it was found that GH affects mitochondrial protein synthesis. Injection of human GH (hGH) to intact and hypophysectomized (hypox) rats revealed that the mitochondrial protein synthetic capacity of liver, measured by radioactive leucine incorporation in vitro as well as in vivo, significantly increased in hGH-treated rats [21]. However, a later study showed that GH is not the only player in mitochondrial biogenesis. Thus, hypox SD rats that were treated with hGH (120 µg/day), triiodothyronine **(**T3, 10 ng/day), or a combination of both hormones for six days showed that cellular respiration was recovered with T3 treatment but not with hGH alone [20].

Identification of the tight relationship between GH and IGF-1 in control of cellular growth and replication led to numerous studies aimed at assessing the effects of IGF-1 on mitochondrial biogenesis. However, as was found with GH, IGF-1 plays indirect effects on mitochondrial biogenesis, likely in synergy with other growth factors. As such, exogenous IGF-1 increased cell volume but not mitochondria in rat sciatic Schwann cells [22]. However, addition of the mitogenic factor neuregulin (NRG) together with IGF-1 increased mitochondrial mass and DNA replication. In MCF-7 and ZR75.1 breast cancer cells, IGF-1 induced the expression of PGC-1β and PGC-1α-related coactivator (PRC), which are required for mitochondrial biogenesis (Figure 2) [13]. Increased mitochondrial mass in these cells associated with reduced expression of the mitophagy mediators BNIP3 and BNIP3L (BCL2/adenovirus E1B 19 kDa protein-interacting protein 3 and L) and impaired mitophagy suggest that IGF-1 indirectly regulates mitochondrial turnover via BNIP3. Increased levels of PGC-1α, Nrf1, Cyt c, and citrate synthase activity were also seen in gastrocnemius muscle cells of old animals treated with GH, likely mediated by IGF-1 (Figure 2) [23]. Age-induced mitochondrial dysfunction in reproductive organs causes reductions in oocyte quality leading to infertility. GH partially rescued the age-induced loss of ovulation stimulation, oocyte formation, and maturation through its effects on mitochondria [24]. Treating young (4 weeks) and aged (32 weeks) female mice with low, medium, or high doses of hGH for 8 weeks resulted in increased oocyte number as well as maturation in aged females that was similar to young females, likely via IGF-1. However, in the latter study, despite these oocyte functional improvements, there was no change in mitochondrial DNA copy number, suggesting again that mitochondrial biogenesis was not affected directly by hGH treatment.

## 3. GH/ IGF-1 Effects on Mitochondrial Respiration and ATP Production During Aging

Mitochondrial function involves several processes, including cellular respiration, energy production via the tricarboxylic acid (TCA) cycle coupled with OXPHOS, calcium homeostasis, cellular replication, apoptosis, and generation of (and protection from) reactive oxygen species (ROS). These functions are fundamental during growth and development and play major roles during aging. Although age-induced mitochondrial dysfunction and overall reductions in the secretion and action of GH and IGF-1 coincide, a clear causality between both processes has not yet been established.

Congenital (life-long) reductions in the activity of the GH/IGF-1 axis was associated with increased longevity in mice [25], flies [26], and nematodes [27,28]. In the long-lived Ames dwarf mice, the activity and expression of several complexes of the electron transport chain (ETC) in liver and in kidney increased [29]. Interestingly, high activity of complex IV of the ETC in Ames mice was in agreement with enhanced oxygen metabolism found also in the long-lived GH receptor null (GHRKO) mice [30]. Elevated expression of key proteins of the mitochondrial respiration complexes in Ames mice coincided with reduced production of H_2_O_2_ in state 3 (ATP production) and state 4 (reflecting mitochondrial leak) of respiration in liver mitochondria [31]. Accordingly, the long-lived GHRKO and Ames mice exhibit improved glucose homeostasis and energy metabolism evidenced by decreased respiratory quotient (RQ) and increased oxygen consumption (VO_2_). In contrast, bGH mice, with high GH/IGF-1 levels show decreased VO(2) and increased RQ [30,32,33]. Taken together, these animal models suggest that diminished GH/IGF-1 activity improves mitochondrial flexibility and increases the capacity for fat oxidation. Interestingly, a recent study found that housing the GHRKO mice at thermoneutral temperature (30 °C) resulted in decreased expression of thermogenic genes in brown adipose tissue (BAT) and elevated core body temperature [32]. However, these mice still maintained their extended longevity phenotype at 30 °C [32], suggesting an intrinsic advantage of mitochondrial function in the GHRKO mice. Together, congenital decreases in the GH/IGF signaling in genetically modified mice were associated with reduced levels of ROS, upregulated activity of the mitochondrial ETC, and overall enhanced mitochondrial function [31,34].

In humans, GH and IGF-1 levels peak during puberty, decline during aging [35], and coincide with unfavorable effects on mitochondrial metabolism in brain (cognition [36]), muscle (sarcopenia [2]), and skeletal tissue (osteopenia [1]). IGF-1 haploinsufficiency (IGF-1^+/−^) in mice was associated with mitochondrial dysfunction accompanied by increased level of lipid peroxidation, protein carboxylation, and intramitochondrial ROS in hepatocytes [37]. The reductions in mitochondrial membrane potential and OXPHOS in the IGF-1^+/−^ mice were resolved with IGF-1 treatment. IGF-1 administration to old Wister rats (103 weeks) for 30 days rescued mitochondrial membrane potential, oxygen consumption rate, proton leak, ATP production, cytochrome oxidase, and ATPase complexes activities in isolated mitochondria from livers [38]. Additionally, the age-associated increases in mitochondrial ROS production, reduced antioxidants activities, and increased apoptosis in isolated mitochondria were resolved by IGF-1 treatment, suggesting cytoprotective effects of IGF-1.

IGF-1 plays essential roles in the function of mitochondria in the central nervous system [39]. Hippocampal mitochondria of 18-month-old mice with adult-induced liver IGF-1 deficiency (iLID) (at 5 months of age) showed lower level of OXPHOS and increased mitochondrial uncoupling, lower level of ATP production, and increased level of oxidative damage, as compared to aged controls [39]. This compromised mitochondrial function in the iLID mice manifested as impaired spatial acquisition and reversal learning. Similarly, specific ablation of IGF-1R in astrocytes [40] caused impaired mitochondrial energy metabolism, OXPHOS, and decreased glucose and amyloid β uptake. Overall, data suggest that increases in IGF-1 signaling in astrocytes may rescue from age-related mitochondrial dysfunction and cognitive decline.

Sarcopenia, age-associated reductions in skeletal muscle-mass, is thought to result from reduced level of GH. Low doses of GH administration to 22-month-old Wistar rats for 8 weeks increased circulating IGF-1 levels, enhanced the synthesis of mitochondrial proteins and antioxidant enzyme activities (catalase, glutathione peroxidase, and glucose-6-phosphate dehydrogenase (G6PDH)), and reduced oxidative damage (measured by the levels of 8-OHdG) in the skeletal muscle [23]. GH administration induced the activation of the anabolic AKT, mTOR, p70S6K, and Myf-5 factors while inhibiting p21, p38, and muscle RING finger-1 (MuRF-1) catabolic signals. Similarly, it was reported that a decline in GH/IGF-1 signaling in muscles of old rats associated with mitochondrial dysfunction. Following treatment with antioxidants, GH and IGF-1 levels in serum increased and associated with improvement of cristae structure and clustering of muscle mitochondria [41]. Likewise, IGF-1 alleviated dysfunctional mitochondria of cardiomyocytes from obese mice [42]. Glucose uptake, ATP production, and aconitase activity increased while lipid peroxidation, ROS production, protein carbonyl content, and apoptosis decreased in transgenic mice with overexpression of cardiomyocyte-specific IGF-1 that were fed a high fat diet. Furthermore, cardiomyocyte-specific IGF-1 induced the expression of Cyt c, PGC-1α, and UCP2 as well as the essential intracellular Ca^2+^ regulatory proteins SERCA2a and the Na^+^/Ca^2+^ exchanger.

Mitochondrial dysfunction was also reported for cortical bone osteocytes in GHRKO mice, which exhibited reduced mitochondrial membrane potential, decreased ATP production, and reduced maximal respiration in both young and old mice [43].

Overall, it seems that GH/IGF-1 signaling involvement in mitochondrial function is multifaceted, with important tissue-, organ-, and age-dependent features. Importantly, there are fundamental differences between the effects of age-induced reductions in GH/IGF-1 on mitochondrial function and those of life-long, congenital, ablations of members of the GH/IGF-1 axis on mitochondrial function.

## 4. GH/IGF-1 Effects on Oxidative Stress During Aging

**Figure 3:**
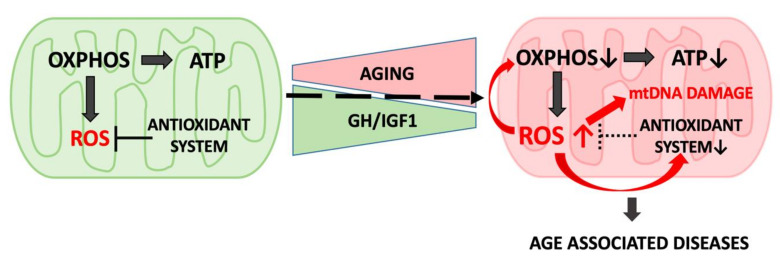
Mitochondria produce ATP and reactive oxygen species (ROS) as byproducts of oxidative phosphorylation (OXPHOS): In youth, ROSs are neutralized by the antioxidant system. Accumulation of proteins and enzymes damaged by escaped ROS leads to impairment of mitochondrial function during aging. Mitochondrial dysfunction is correlated with the decline in GH/IGF1 signaling and is linked to a variety of age-related diseases.

According to the free radical theory of aging, ROSs generated by the ETC in the mitochondria or via nitric oxide metabolism in the cytosol have the potential to result in oxidative damage to DNA, proteins, and lipids and thus to accelerate aging (Figure 3) [44,45,46,47]. Superoxide anion (O_2_^−^) and H_2_O_2_ are produced in mitochondria as byproducts of OXPHOS. Further, H_2_O_2_ can be converted into a dangerous hydroxyl radical (HO) during Fenton’s reaction in the presence of Fe^2+^ [48]. Antioxidant defense includes enzymatic activation of superoxide dismutases (SODs), which are metalloproteins that convert superoxide to hydrogen peroxide and molecular oxygen. There are three types of SODs: Cu/Zn-SOD, predominantly located in the cytosolic fractions; Mn-SOD, located in the mitochondria; and EC-SOD, which is found in the extracellular space [49]. Catalase is a heme protein located predominantly in peroxisomes and the inner mitochondrial membrane.

There are numerous reports showing that GH/IGF-1 signaling controls the expression and activity of antioxidant enzymes and thus regulates the level of oxidative stress. Congenital IGF-1R haploinsufficiency in mice (*Igf1r+/−*) alters sensitivity to oxidative stress. Embryonic fibroblasts from *Igf1r+/−* mice are more resistant to hydrogen peroxide-induced cell death [25]. Accordingly, we found that IGF-1 null mice that exclusively express hepatic IGF-1 transgene (KO-HIT mice) [50] show increased levels of lipid peroxidation products in serum and increased mortality rate at 18 months of age in both sexes, suggesting that elevations in serum IGF-1 are harmful. Mutations that affect pituitary development (*Prop1* and *Pit1*) and consequently lead to decreases in GH, thyroid stimulating hormone, or prolactin are associated with resistance to oxidative stress. Ames dwarf mice have very low serum IGF-1 levels and increased activities of catalase and Cu/Zn SOD [51]. These mice show reduced levels of DNA and protein oxidation in liver [52] and reduced serum and liver F2-isoprostanes, which are a stable lipid peroxidation product [53]. Similarly, the GH-deficient Snell and *lit/lit* dwarfs show resistance to oxidative stress [54,55]. Supplementation of GH to Ames dwarf mice for 7 days increased plasma IGF-1 levels and body and liver weights. However, mitochondrial glutathione S-transferase (GST) proteins (GSTK1 and GSTM4) significantly reduced with treatment [56,57]. Furthermore, glutaredoxins, which are localized in the mitochondria and sense cellular oxidative stress, significantly reduced with GH administration. In line with these data, it was reported that GHRKO mice exhibit increases in glutathione (GSH) and methionine (MET) metabolism in several tissues, making them more resistant to oxidative damage and delayed aging [58]. However, GHRKO mice, although resistant to oxidative stress, do not show improved free radical scavenging in the liver or kidney [59], though it is possible that other tissues such as muscle respond differently. Although informative, data from congenital models of the GH/IGF-1 axis cannot be directly extrapolated to normal aging in other animal models or in humans. These models exhibit altered developmental programming that affect their aging.

GHRKO mice resemble the human Laron syndrome (LS), which is caused by a deletion or an inactivating mutation of the *GHR* gene [60,61,62,63]. Similar to the GHRKO mice, LS patients have short stature, increased body adiposity, and low IGF-1 in serum [63,64]. Genome-wide microarray studies conducted on lymphocytes from LS patients identified a series of genes that are differentially expressed in various pathways, including oxidative stress, apoptosis, metabolism, Jak-STAT, and PI3K-AKT signaling. Among the overexpressed genes in LS, thioredoxin-interacting protein (*TXNIP)* was identified as a new target for IGF-1 and insulin action. TXNIP belongs to the α-arrestin family [65]. It binds to the catalytic active-center of reduced thioredoxin (TRX) and inhibits its expression and activity, highlighting the key role of TXNIP in redox regulation. Oxidative stress leads to TXNIP shuttling from the nucleus into the mitochondria. TXNIP inhibits proliferation via activation of the apoptosis signal regulating kinase 1 (ASK1) [66] and functions as a tumor suppressor, being commonly silenced in cancer cells [67,68,69,70,71]. Similar to LS patients, GHRKO mice show reduced tumor incidence in experimental models of cancer [72]. In accordance with its enhanced expression in LS-derived cells, qPCR revealed that *TXNIP* expression increased approximately two-fold in livers of GHRKO mice, while it decreased in HIT mice overexpressing IGF-1 in the liver.

**Figure 4:**
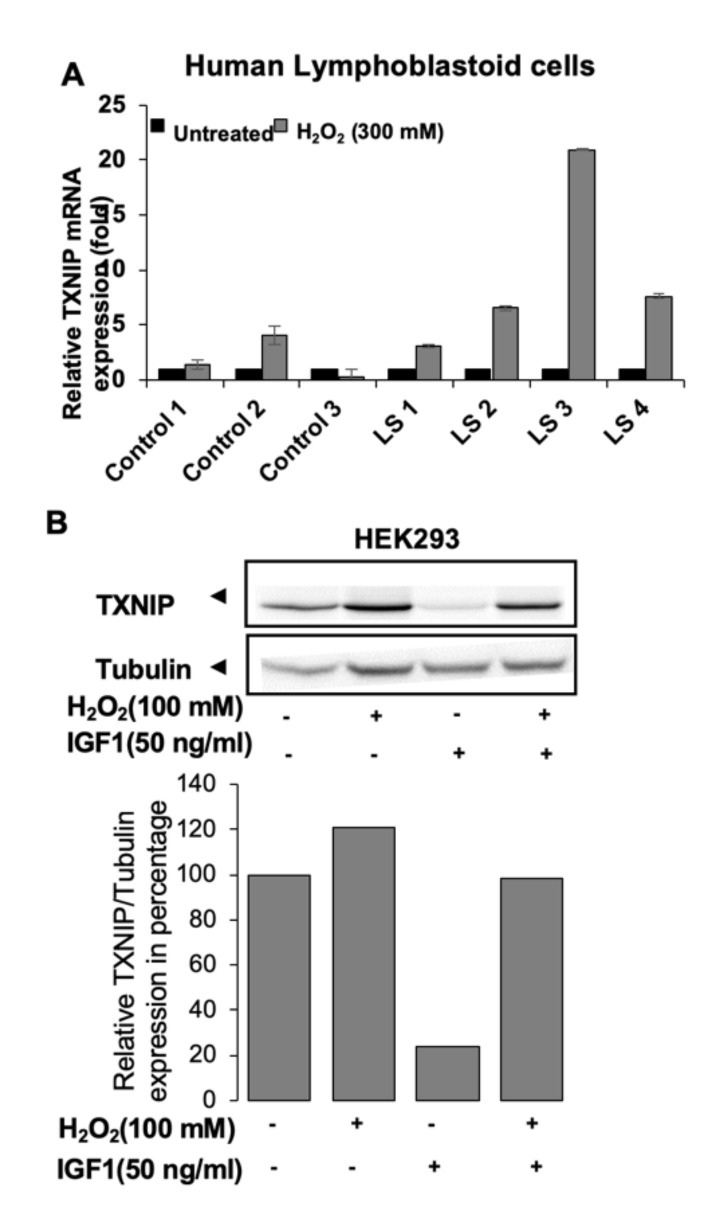
Effect of oxidative stress on thioredoxin-interacting protein (TXNIP) levels [79]: (**A**) Effect of oxidative stress on TXNIP levels in Laron syndrome (LS)-derived and control lymphoblastoids. Four individual LS and three control lymphoblastoid cell lines were treated with 300 mM of H_2_O_2_ for 2 h, and levels of TXNIP mRNA were measured by RT-QPCR. A value of 1 was given to TXNIP mRNA levels in untreated cells (solid bars). (**B**) Serum-starved HEK293 cells were treated with H_2_O_2_ (100 mM) or IGF1 (50 ng/mL) or both for 2 h. TXNIP and tubulin were detected by Western blotting.

LS lymphocytes were shown to display protection from oxidative stress [73]. Accordingly, induction of oxidative stress in lymphocytes from LS patient (Figure 4A) leads to upregulation of *TXNIP*. The capacity of IGF-1 to downregulate the oxidative stress-induced TXNIP upregulation (Figure 4B) indicates that IGF-1 could rescue the cells by downregulating TXNIP. TXNIP acts as an oxidative stress mediator by inhibiting TRX activity or by limiting its bioactivity [74]. The redox-related protein complex TRX/TXNIP, or “redoxisome,” is a critical regulator of ROS signalling and is involved in the pathogenesis of various diseases, including autoimmune and degenerative conditions [68]. The finding that TXNIP levels are increased in response to oxidation in LS patient-derived but not control lymphoblastoid cells is of major translational relevance.

**Figure 5:**
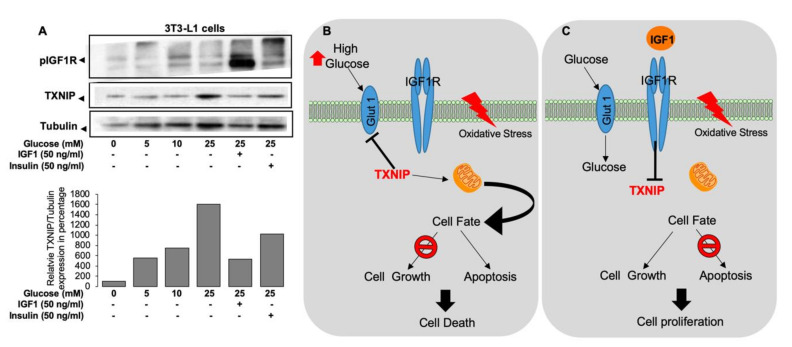
Effect of glucose stress on TXNIP levels [79]: (**A**) Serum-starved 3T3-L1 cells were maintained in medium with different concentrations of glucose in the presence or absence of IGF1 or insulin for 6 h. TXNIP, phospho-IGF-1R, and tubulin were detected by Western blotting. Schematic representation of the regulation of TXNIP by IGF1 signaling: (**B**) Under normal serum-free conditions, TXNIP is upregulated upon oxidative and glucose stresses. The activated TXNIP inhibits glucose uptake and is capable of mediating mitochondrial mediated apoptosis. (**C**) Upon IGF1 stimulation, TXNIP is downregulated even under oxidative and glucose stresses. Suppression of TXNIP leads to inhibition of apoptosis with ensuing increase in cell proliferation.

Apart from oxidative stress, TXNIP was reported to function as a strong glucose sensor as its expression increased upon high-glucose stress [75]**.** *TXNIP* knockout mice (*TXNIP* KO) show impaired metabolic homeostasis, including adipogenesis and reduced gluconeogenesis [76], and decreased glucose uptake [77]. Similarly, a recently described human mutation demonstrated that diminished TXNIP function is linked to inefficient utilization of glucose [78]. Accordingly, glucose stress (hyperglycemia) upregulated TXNIP levels in 3T3LY adipocytes and was downregulated by IGF-1 and insulin (Figure 5A) [79]. Overall, these studies show that oxidative and glucose stresses induced TXNIP levels at both the transcriptional and translational levels (Figure 5B) and that IGF-1 indirectly regulates cellular TXNIP, protecting the cells from apoptosis (Figure 5C) [79]. The potential involvement of epigenetic mechanisms, particularly DNA methylation and histone acetylation, in inhibitory regulation of *TXNIP* gene expression by IGF-1 is currently unknown.

Notably, there are numerous studies indicating that administration of GH/IGF-1 in vivo or in vitro protects from oxidative stress, specifically during aging. Old Wistar rats treated with GH showed increased circulating IGF-1 levels and reductions in age-associated oxidative stress in skeletal muscle [23]. This was accompanied by increased levels of the antioxidant enzymes catalase, glutathione peroxidase, and G6PDH. Similarly, supplementation of IGF-1 to aged rats associated with reduced oxidative damage and restored levels of SOD, glutathione peroxidase, and catalase in hippocampus [34]. Hepatic tissue level of catalase was also restored with IGF-1 treatment in old rats, suggesting antioxidant properties of IGF-1 in brain and liver [34].

IGF-1 is reported to be protective against oxidative stress in cardiac and skeletal muscle. Pathologic left ventricular remodeling and functional loss following myocardial infarction were more severe in dwarf rats (*dw/dw*) with significantly reduced GH/IGF-1 [80]. Using an ex vivo murine model of myocardial ischemia/reperfusion injury, it was found that IGF-1 protects ischemic myocardium from further reperfusion injury, likely via maintenance of mitochondrial to nuclear DNA ratio within heart tissue [81] and by activation of the PI3K-Akt and/or Erk 1/2 kinase cascades [82]. Likewise, hearts of IGF-1 transgenic mice were protected from ischemia and reperfusion [83], and streptozotocin-induced diabetic cardiomyopathy in mice resulted in accumulation of nitrotyrosine (a reactive oxygen product) in vivo and the formation of H_2_O_2_ in myocytes in vitro that were rescued in IGF-1 transgenic mice [84]. Additionally, IGF-1 protected from 2:4 dinitrophenol-induced oxidative stress in rat muscle in vitro [85] and myoblasts protected from H_2_O_2_ stress-induced apoptosis [86].

The protective effects of the GH/IGF-1 axis from oxidative stress was demonstrated for many cell types. Endothelial cells from Ames dwarf mice show elevated levels of H_2_O_2_, increased mitochondrial ROS, and decreased antioxidative enzymes such as SODs and glutathione peroxidase. These observations were in accordance with in vitro findings using cultured aortic segments and human coronary arterial endothelial cells. Treatment of these cultures with GH and IGF-1 lead to elevations in the level of antioxidants and reduced prooxidant levels [87]. In astrocytes, downregulation of the IGF-1R increased mitochondrial ROS production and reduced resistance to external oxidative damage [40]. IGF-1 promoted the survival of rat primary cerebellar neurons and of immortalized hypothalamic rat GT1-7 cells following H_2_O_2_-induced oxidative stress [88]. There is a long list of studies utilizing numerous cell types, showing the protective effects of GH/IGF-1 from oxidative stress that, although important, were not included in our review.

Antioxidant administration to old animals can elevate circulating GH and IGF-1 levels. Thus, treatment of Wistar rats or senescence-accelerated OXYS rats with antioxidants and, in particular, with mitochondrial antioxidants prevented the age-associated decrease in serum levels of GH and IGF-1 [41,89]. These were accompanied by improvements in pathologies such as retinopathy and cataract, learning ability and memory, and immune system decline [89,90]. Treatments of aged rats with mitochondrial antioxidants also led to improvement of mitochondrial structure-disorganization developed with age in muscle tissues. Disorders of muscles tissue mitochondrial apparatus in old rats could be driven by a decline in GH/IGF-1 signaling, as improvement of cristae structure and clustering of muscle mitochondria was correlated with an increase of GH and IGF-1 levels in serum after treatment with mitochondrial antioxidants [41].

In summary, insufficient levels of GH/IGF-1 are associated with organ-specific impairment of free radical scavenging systems. GH/IGF-1 plays significant roles in regulating oxidative stress, which is clearly only one of many mechanisms affecting mitochondrial function during aging. Finally, there are fundamental differences in handling oxidative stress between age-associated decline in GH/IGF-1 (somatopause) and congenital impairments in the GH/IGF-1 axis.

## 5. GH/IGF-1 Effects on Cellular Senescence

Cellular senescence is characterized by an irreversible block of the cell cycle. This mechanism was initially thought to function as protection against cancer [91], but later studies have found that senescence is linked to aging and age-related diseases. Senescent cells undergo numerous phenotypic and metabolic modifications. They show increased cells size [92], dysfunctional mitochondria and telomeres, impaired DNA damage response, increased secretory functions, formation of heterochromatic foci, and a senescence-associated secretory phenotype (SASP). In fact, the SASP is a main feature of senescent cells, which secrete pro-inflammatory cytokines and chemokines, growth factors, and proteases, forming a toxic and harmful microenvironment to non-senescent cells [93,94,95,96,97].

Mitochondria undergo drastic changes in morphology and function in senescent cells. Given the complex functions of mitochondria, it is hard to dissect the specific mitochondria-mediated mechanisms leading to cell senescence. Several potential mechanisms, mediated by mitochondria, that may lead to cell senescence include increased ROS production and elevations in damage-associated molecular patterns (DAMPs) leading to DNA damage response (DDR), which locks the cells in a senescence mode, leading to SASP. Senescent cells exhibit increased mitochondrial mass but, at the same time, show reduced OXPHOS and rely mainly on ATP from glycolysis. Increased mitochondrial mass is driven by upregulation of PGC-1β [98]. Accordingly, deletion of PGC-1β in mice delayed several aspects of senescence [98]. In line with these data, it was shown that senescent pancreatic beta cells exhibit increased mitochondrial biogenesis [99].

Pituitary adenomas expressing and secreting GH exhibit a senescent phenotype. Autocrine/paracrine GH acts in pituitary cells as an apoptosis switch for p53-mediated senescence, likely preventing the pituitary adenoma cells from progression to malignancy [100]. Skin fibroblasts from acromegalic patients (with excess GH and IGF-1) exhibit shortened telomeres and cellular senescence [101]. Similarly, senescence-associated gene expression of p16 and IL-6 increased in white adipose tissue of 10-month-old female bGH transgenic mice as compared to controls. In addition, β-galactosidase (β-gal)-positive cells (senescence-associated) were elevated in GH-injected 19-month-old female mice as compared to age-matched saline-injected controls [102]. Accordingly, mice with a specific deletion of the IGF-1R in cardiomyocytes show delayed development of aging-associated myocardial pathologies [103]. Cultured cardiomyocytes treated with IGF-1 exhibited increased senescence, while inhibition of phosphoinositide 3-kinase prevented the IGF-1-mediated increase in interleukin (IL)-1α, IL-1β, receptor activator of nuclear factor-κB ligand, and p21 protein levels [103]. Finally, prolonged IGF-1 treatment of MCF7 cells inhibited SIRT1 deacetylase activity, associated with increased p53 acetylation and activation, and lead to premature cellular senescence [104]. Similarly, prolonged exposure of primary human mesangial cells to glycated albumin (GA) was associated with IGF-1 release, activation of the IGF-1R, and enhanced cellular senescence. GA-induced IGF-1R activation associated with a reduction in the catalase content likely through activation of the Ras and Erk1/2 pathway. Downregulation of the IGF-1R via overexpression of klotho lowered p53 and reversed the senescence phenotype [105]. In vitro, cell senescence is often observed once cells reach confluency. IGF-1 promoted senescence in mouse embryonic fibroblasts and human fibroblasts as well as in rat vascular smooth muscle cells (VSMCs) after attaining confluency. The IGF-1-induced senescence in these cells was associated with elevated cellular ROS, p53, and p21 protein levels and the DNA damage marker γH2AX [106].

On the other hand, there are studies showing that GH/IGF-1 protects from senescence. GH treatment (0.4 mg/d for 7 days) of endothelial progenitor cells from patients with atherosclerosis reversed age-related dysfunction and attenuated senescence (indicated by increased telomerase activity) [107]. These findings were in line with another study in which GH was incorporated into reconstituted high-density lipoprotein (rHDL) and delivered to zebrafish. In this study, GH enhanced anti-atherosclerotic activity and antisenescence activity with inhibition of fructose-mediated glycation [108]. Likewise, IGF-1 gene transfer to CCl_4_-treated rats (with liver injury and fibrosis) relieved hepatocyte oxidative stress and premature senescence, likely mediated by the p53/progerin pathway [109]. In vitro, IGF-1 treatment of H_2_O_2_-exposed hepatocytes reversed oxidative stress-induced premature senescence via enhancing cytoplasmic AKT1–p53 interaction and by subsequently inhibiting nuclear p53–progerin interaction [109].

## 6. GH/IGF-1 Effects on Mitochondria-Mediated Apoptosis During Aging

Similar to senescence, programmed cell death, or apoptosis, is believed to be a consequence of cellular stress and mitochondrial dysfunction. However, the roles of apoptosis during aging are uncertain. In general, the determinants of whether a cell activates senescence or apoptosis pathways are cell-type, stress-type, and stress-intensity dependent. It is important to note that apoptosis occurs mainly through activation of a mitochondrial intrinsic pathway, which depends on caspase 9 and apoptotic protease activating factor 1 (Apaf1) or via an extrinsic pathway mediated by the Fas ligand and tumor necrosis factor receptor (TNFR) and by activation of caspase 8. Herein, we will describe shortly the roles of the GH/IGF-1 axis in mitochondrial (intrinsic)-mediated apoptosis.

**Figure 6:**
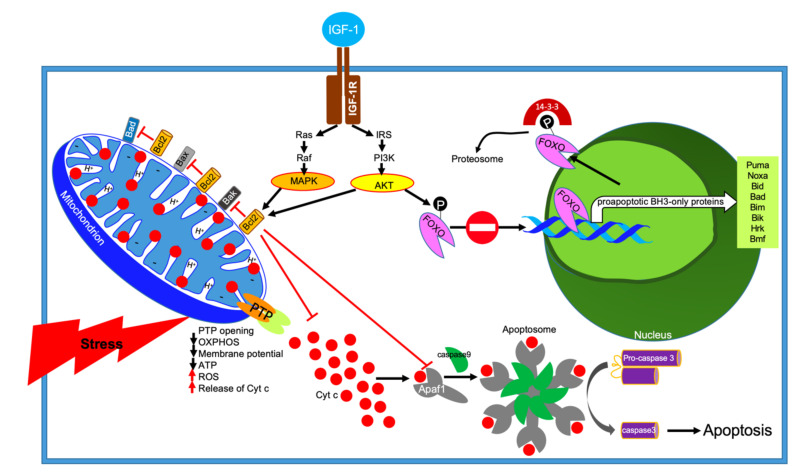
The effects of IGF-1 on mitochondria-mediated apoptosis: Cellular stress leads to permeability transition pore (PTP) opening, reduction in OXPHOS function, membrane potential, and ATP production, while ROS production is increased, altogether leading to cyt c release. Cytosolic Cyt c activates the apoptosome, leading to cell death. IGF-1R-mediated inhibition of apoptosis occurs via activation of two major signaling pathways that trigger phosphorylation cascade of cytosolic and nuclear proteins regulating transcription and activation of proteins involved in protection from apoptosis. The AKT pathway leads to phosphorylation of Forkhead box transcription factors of the class O (FOXO) proteins and subsequent inhibition of their transcriptional activity. The MAPK pathway, stimulated by IGF-1 binding, activates the Bcl antiapoptotic family of proteins.

A key step in mitochondria-mediated apoptosis is the release of Cyt c from the inner mitochondrial space (Figure 6). Several pathways are known to trigger Cyt c release including opening of the mitochondrial permeability transition pore (PTP). Opening of the PTP leads to mitochondrial swelling, depolarization of the membrane potential, subsequent rupture of the outer membrane, and nonselective release of intermembrane space proteins. Once released, Cyt c binds to Apaf1 and activates a group of cysteine proteases called caspases that cleave an array of substrates and proteins that are vital to cellular function. The Apaf1-cyt c-caspase 9 form apoptosome complexes. Apoptosome, central to the apoptotic pathway, binds to other pro-enzymes and cleaves them to their active forms.

Cyt c release from mitochondria is a primary signal for B-cell lymphoma 2 (Bcl-2)-regulated inhibition of apoptosis [110,111]. Bcl-2 is localized in the outer mitochondrial membrane (Figure 6). Bcl-2 belongs to a large family of proteins with antiapoptotic properties (i.e., Bcl-XL, Bcl-w, Mcl-1, A1, Bcl-Rambo, Bcl-L10, and Bcl-G) and proapoptotic properties (i.e., Bax, Bak, and Bok) [112]. There are several other proteins that block the antiapoptotic activity of Bcl-2, which are termed proapoptotic BH3-only proteins (i.e., Puma, Noxa, Bid, Bad, Bim, Bik, Hrk, and Bmf) [113]. However, it appears that Bax/Bak are the key regulatory targets where many intracellular signals converge and determine a cell’s fate [114]. It was widely accepted that the role of the antiapoptotic BCL-2-like proteins is to inhibit their proapoptotic counteracting partners, such that the balance between anti- and proapoptotic BCL-2 family proteins determines cell fate. However, this model is oversimplified in view of later discoveries, including the occasional interconversion of anti- and proapoptotic activities of these proteins, and the findings that BCL-2 family members exhibit nonapoptotic functions [115,116].

The insulin and IGF-1 signaling pathway (IIS) are the most evolutionarily conserved pathway of aging. IGF-1 has been recognized as a survival factor of numerous cell types. Activation of the insulin or IGF-1 receptors elicits activation of several pathways; among them are the mitogen-activated protein kinase (MAPK) pathway and the PI3K-AKT pathway. Activation of AKT leads to phosphorylation and inactivation of a group of forkhead box transcription factors of the class O (FOXO) factors by retaining them in the cytoplasm. Phosphorylation of FOXOs and their binding to the regulatory proteins 14-3-3 sequester them from the nucleus, leading to suppression of FOXO-dependent transcription (mostly the BH3-only proteins) [117,118,119]. Thus, the survival factor IGF-1 attenuates apoptosis via activation of the PI3K-AKT pathway and inhibition of FOXO proteins.

FOXOs are the most important transcriptional effectors of the IIS, and as such, they are tightly regulated post-translationally [120]. Mammals have four FOXO genes: FOXO1, FOXO3, FOXO4, and FOXO6. FOXO1-null mice are embryonically lethal, while FOXO3, FOXO4, and FOXO6 show mild phenotypes [121]. Tissue-specific approaches to delete FOXO proteins 1, 3, and 4 have established the importance of these factors in regulation of cell fate. For example, ablation of Foxo1, 3, and 4 proteins in osteoblasts associated with increases in oxidative stress and osteoblast apoptosis [122], while osteoblast-specific overexpression of Foxo3 decreased oxidative stress and apoptosis.

In summary, the GH/IGF-1 axis regulates mitochondrial (intrinsic)-mediated apoptosis mostly via activation of the PI3K-AKT/FOXO pathway and possibly via cell-specific transcriptional regulation of antiapoptotic genes. However, while during development and adult stages apoptosis is required for the normal cell turnover in specific tissues (such as endothelium, intestinal epithelium, etc.), the role of apoptosis during aging is not clear. In fact, apoptosis was shown to decrease in adipose mesenchymal stem cells from healthy young, middle-aged, and aged volunteers [123]; in bone marrow mesenchymal stem cells from old mice [124]; in livers of old rats challenged with DNA-damaging agents as compared to their young controls [125]; and even in response to radiation of peripheral blood lymphocytes from old mice [126]. Accordingly, reductions in markers of apoptosis during normal aging was also found in human serum [127]. Perhaps during aging, mitochondrial dysfunction leads mostly to cell senescence as opposed to apoptosis.

## 7. GH/IGF-1 Effects on Mitochondrial Function During Inflammation

Inflammation is a defense mechanism against harmful stimuli that damage cellular homeostasis [128]. Inappropriate response to cellular damage may lead to chronic inflammation, including autoimmune diseases, diabetes, allergies, cardiovascular diseases, asthma, cancer, arthritis, and aging [129,130]. Response to various immunological challenges entails particular metabolic configurations for energy generation required for biosynthesis of molecules [131]. Dysfunctional mitochondria with excessive ROS, abnormal calcium and potassium ion mobilization, and unusual ATP or NAD^+^ levels can trigger immune response [132]. On the other hand, inflammatory cytokines acting upon cells can trigger intracellular signaling cascades altering mitochondrial dynamics and mitophagy and eventually resulting in cell death [133,134].

The GHR and IGF-1R are expressed on immune cells and participate in thymic development and differentiation of immune cells such as T-cell, B- cell, and natural killer cells as well as in antigen presentation, antibody production, etc. [135,136]. Additionally, there are studies showing local production of GH and IGF-1 by lymphocytes, suggesting an autocrine/paracrine regulation of immune cells [137,138]. Further, human lymphocytes treated with super physiological levels (abuse) of IGF-1 can undergo cytoskeletal reorganization and overproduction of cytokines, augmenting the inflammatory response [139]. On the other hand, children with chronic inflammatory disorders exhibit excessive pro-inflammatory cytokines production and show growth failure and pubertal abnormalities [140]. Similarly, the elderly, with reduced GH or IGF-1 signals show poor response to immune stimuli [141].

The effects of GH/IGF-1 on mitochondria of inflammatory cells are underexplored. Aging is often associated with metabolic abnormalities accompanied by increased chronic inflammation in many tissues, including the brain. In fact, GH signaling was found to positively modulate brain inflammation in aged mice [142]. Accordingly, GHRKO mice showed reduced inflammation in the hypothalamus. GHRKO and Ames mice displayed lower glial fibrillary acidic protein (GFAP) and tumor necrosis factor (TNF)-α levels in the hypothalamus, indicative of reduced inflammation. Consequently, administration of GH to GHRKO and Ames mice led to increased GFAP and TNF-α and increased brain inflammation [142]. As mentioned above, the GHRKO is a mouse model with congenital ablation of the GH/IGF-1 axis. It is unclear whether this axis plays a role in systemic or central inflammation, particularly during natural aging where a gradual fall in GH/IGF-1 signals happens.

## 8. Summary

GH and IGF-1 are pleotropic hormones affecting multiple cellular functions, including cell proliferation, differentiation, metabolism, and cell survival. Both hormones activate many signaling cascades implicated in regulation of mitochondrial proteins expression and function. Most evidence indicates that the effects of GH on mitochondrial mass and function are indirect and mostly mediated by IGF-1. IGF-1 affects mitochondrial mass via increased transcriptional activities of key factors involved in mitochondrial biogenesis such as PGC-1α. Additionally, it appears that the effects of IGF-1 on mitochondrial respiration are indirect and coincide with enhanced synthesis of mitochondrial proteins such as Cyt c and UCP. With respect to oxidative stress, the literature is divided and studies showing either positive or negative effects of GH or IGF-1 have been published. While congenital mouse models with life-long decreases in GH/IGF-1 axis signaling indicate mostly protection from oxidative stress, models of age-induced decreases in GH/IGF-1 signals, as seen in humans, associate with increased oxidative stress.

Finally, it is widely accepted that GH/IGF-1 are involved in cell senescence and apoptosis. The molecular mechanisms involved in GH/IGF-1-mediated cellular senescence are still poorly understood. Both hormones exert a dual function and promote, on one hand, cell proliferation and, on the other hand, cellular senescence. Therefore, it is conceivable that the dose and duration of GH/IGF-1 exposure might regulate senescence, and that the effects of GH/IGF-1 on senescence are tissue- and cell type-specific. On the other hand, the protective roles of IGF-1 from mitochondrial-mediated apoptosis have been better defined. Studies of numerous cell types and animal models have shown that IGF-1-mediated activation of the PI3K-AKT/FOXO pathway upregulates transcription of antiapoptotic genes.

## Abbreviations

iLID	Adult-induced liver IGF-1 deficiency
ALS	Acid labile subunit
ATP	Adenosine triphosphate
ASK1	Apoptosis signal regulating kinase 1
Bcl-2	B-cell lymphoma 2
β-gal	β-galactosidase
bGH	Bovine growth hormone
BNIP3 and BNIP3L	BCL2/adenovirus E1B 19 kDa protein-interacting protein 3 and L
BAT	Brown adipose tissue
CS	Cockayne syndrome protein
Cyt c	Cytochrome c
DAMPs	Damage-associated molecular patterns
DDR	DNA damage response
ETC	Electron transport chain
FOXO	Forkhead box transcription factors of the class O
GH	Growth hormone
GHR	Growth hormone receptor
GHRKO	GH receptor null
GHRH	GH-releasing hormone
GHRHR	GH-releasing hormone receptor
G6PDH	Glucose-6-phosphate dehydrogenase
GST	Glutathione S-transferase
GSH	Glutathione
GA	Glycated albumin
HO	Heme oxygenase
HIT	Hepatic IGF-1 transgene
hGH	Human GH
H_2_O_2_	Hydrogen peroxide
hypx	Hypophysectomized
IGF-1	Insulin-like growth factor-1
IGFBPs	IGF-binding proteins
IGF-1R	Insulin-like growth factor-1 receptor
IIS	Insulin and IGF-1 signaling pathway
IL	Interleukin
JAK2	Janus kinase 2
LS	Laron syndrome
MET	Methionine
mtDNA	Mitochondrial genome
MuRF-1	Muscle RING finger-1
ND1 through ND6	NADH dehydrogenase of complex I
TNF	Necrosis factor
NRG	Neuregulin
NO	Nitric oxide
NRF1 and 2	Nuclear respiratory factors
OXPHOS	Oxidative phosphorylation
PTP	Permeability transition pore
PGC1α	Peroxisome proliferator-activated receptor gamma coactivator 1 α
PPARs	Peroxisome proliferator-activated receptors
PRC	PGC-1α-related coactivator
PI3K/AKT	Phosphoinositide-3-kinase (PI3K)/Protein kinase B (PKB, or Akt)
MAPK	Rat sarcoma GTPase protein (Ras)/Rapidly accelerated fibrosarcoma protein kinase (Raf)/Mitogen-activated protein kinase
ROS	Reactive oxygen species
RQ	Respiratory quotient
rHDL	Reconstituted high-density lipoprotein
SST	Somatostatin
SSTR	SST-receptors
Shc	Src homology 2 domain containing transforming protein
STAT5b	Signal transducer and activator of transcription 5B
SERCA2a	Sarco/endoplasmic reticulum Ca^2+^-ATPase-2a
SASP	Senescence-associated secretory phenotype
TF	Transcription factors
TXNIP	Thioredoxin-interacting protein
TRX	Thioredoxin
T3	Triiodothyronine
TOMs	Transporters located at the outer mitochondrial membrane
TCA	Tricarboxylic acid
TNFa	Tumor necrosis factor-alpha
VSMCs	Vascular smooth muscle cells
